# Low Frequency of Dementia with Lewy Bodies Diagnosis in a Colombian Memory Clinic

**DOI:** 10.1002/mdc3.70345

**Published:** 2025-09-08

**Authors:** Felipe Botero‐Rodríguez, José Manuel Santacruz‐Escudero, Miguel Germán Borda, Salomón Salazar‐Londoño, Carlos Cano‐Gutiérrez, Dag Aarsland, COL‐DLB

**Affiliations:** ^1^ Intellectus Memory and Cognition Center Hospital Universitario San Ignacio Bogotá Colombia; ^2^ Centre for Age‐Related Medicine (SESAM) Stavanger University Hospital Stavanger Norway; ^3^ SynaptIA–Inteligencia artificial para la investigación en salud mental Bogotá Colombia; ^4^ Semillero de Neurociencias y Envejecimiento, Ageing Institute, Medical School Pontificia Universidad Javeriana Bogotá Colombia; ^5^ Departamento de Psiquiatría y Salud Mental Pontificia Universidad Javeriana Bogotá Colombia; ^6^ Department of Neurology Clínica Universidad de Navarra Pamplona Spain; ^7^ Centro de Investigación en Ciencias de la Salud (CICSA), FCS Universidad Anáhuac México Huixquilucan México; ^8^ Centre for Healthy Brain Ageing, Institute of Psychiatry, Psychology, and Neuroscience King's College London London United Kingdom

**Keywords:** Lewy body disease, synucleinopathies, dementia, Latin America, prevalence

## Abstract

**Background:**

The global burden of dementia is increasing, particularly in low‐ and middle‐income countries. Dementia with Lewy bodies (DLB) is the second most common neurodegenerative dementia but remains underreported and frequently misdiagnosed. Its prevalence in Latin America is largely unknown.

**Objective:**

The aim was to determine the frequency of DLB in a large memory clinic in Colombia and compare its clinical presentation with other neurodegenerative conditions, including Alzheimer's disease, frontotemporal dementia (FTD), vascular dementia, and Parkinson's disease dementia (PDD).

**Methods:**

We conducted a retrospective study at a referral memory clinic in Bogotá, Colombia, from January 2018 to December 2022. DLB was identified based on the Fourth Consensus Report criteria. Random samples of patients with other neurodegenerative conditions were selected for comparison, maintaining a maximum ratio of 4:1. Clinical assessments were conducted by an interdisciplinary team using validated neurocognitive, neuropsychiatric, neuropsychological, and functional tools. Imaging and biofluid biomarkers were not available.

**Results:**

Of 5518 patients, 38 (0.6%) were diagnosed with DLB. These individuals were predominantly male, with an average age of 72.3 years. Significant differences were observed between groups in age of onset, motor and cognitive function, and neuropsychiatric symptoms. Hallucinations were more common in DLB and PDD, whereas behavioral disturbances were frequent in FTD and DLB. Core symptoms of DLB were also present in patients diagnosed with other conditions, although cognitive fluctuations were not registered.

**Conclusions:**

DLB is likely underdiagnosed in this setting. Improved recognition and management are essential.

The burden of dementia‐related diseases is increasing, particularly in low‐ and middle‐income countries (LMIC), where prevalence is projected to triple by 2050.^1^ Alzheimer's disease (AD) and dementia with Lewy Bodies (DLB) are the 2 most prevalent neurodegenerative dementias.^2^ DLB is estimated to comprise 3.2% to 7.1% of all diagnosed dementias in incidence studies, with prevalence rates ranging from 0.3% to 24.4% of all dementia cases outside Latin America.^3^ Furthermore, this significant prevalence variation across studies is probably due to misdiagnosis and underdiagnosis,^2^ which is probably related to limited availability, insufficiently trained personnel on identification, and variability in clinical presentation.^4^

Latin America lacks precise reports on dementia, especially regarding DLB.^5^, ^6^ Also, previously published evidence on DLB in Latin America has addressed various topics, including risk factors, neuropsychiatric symptoms, screening tools for cognitive impairment, and cerebrospinal fluid biomarkers.^6^, ^7^, ^8^, ^9^, ^10^ However, research on DLB in this region is hindered by a lack of standardization in clinical evaluations and research methodologies, difficulties in patient recruitment, limited knowledge of the disease, insufficient studies on this population, and underrepresentation in DLB research.^6^, ^11^ In response, the Colombian Consortium for the Study of Lewy Body Dementia (COL‐DLB) was formed in 2020 to address the challenges in DLB diagnosis and bridge the gap.^11^

Due to this challenge, there is a clear need to improve diagnostic accuracy and better identify dementia subtypes potentially linked to DLB. To address this, our study aims to analyze the frequency of DLB diagnoses based on neurological, geriatric, psychiatric, and neuropsychological assessments at the largest memory clinic in Colombia, located in the capital city of Bogotá, Colombia. In addition, we characterize the clinical presentation of DLB with other neurodegenerative conditions such as AD, frontotemporal dementia (FTD), vascular dementia (VaD), and Parkinson's disease dementia (PDD).

## Patients and Methods

We conducted a retrospective cross‐sectional study. The research protocol was approved by the Institutional Research Ethics Committee of San Ignacio University Hospital and Pontificia Universidad Javeriana (act no. 04/2024).

### Population

The study population consisted of 5518 patients who attended the Intellectus Memory and Cognition Center at San Ignacio University Hospital in Bogotá, Colombia, between January 1, 2018, and December 31, 2022. The Intellectus Memory and Cognition Center is the largest memory clinic in Colombia and one of the largest in Latin America, with more than 14,000 patients evaluated from 2012 to 2024. It serves mainly the capital city population of more than 8 million, but it is also a referral center for the whole country. The assessed population includes public and private services.

Through a retrospective analysis of clinical records, we identified patients who had been diagnosed with DLB in the study population. Then, to facilitate comparisons with other dementia etiologies, we randomly paired individuals diagnosed with AD, FTD, VaD, and PDD from the same cohort, as described in the “Statistical Analysis” section. All patients presented with clinical dementia rating (CDR) of >1, so they were diagnosed with dementia.

### Diagnostic Procedures Performed at Intellectus

Every referred patient received a standardized interdisciplinary clinical evaluation conducted by specialists in geriatrics, psychiatry, neurology, and neuropsychology. This assessment includes a comprehensive list of standardized diagnostic tools and rating scales based on specific protocols oriented to the assessment of patients with functional, cognitive, and behavioral problems (see later). Patients' visit works as a circuit for 4 hours, and a rotation system is applied so that each patient passes through each assessment individually. When the assessment is scheduled, patients are asked to take a structural magnetic resonance image and routine blood test to exclude other etiologies (thyroid‐stimulating hormone, vitamin B12, vitamin D, rapid plasma regain for screening syphilis). Functional imaging or fluid biomarkers are available only in exceptional cases due to the difficulty in accessing these paraclinical examinations.

The clinical evaluation involved a comprehensive interdisciplinary approach: geriatricians assessed global medical health, social circumstances, and functional status; psychiatrists identified neuropsychiatric symptoms and mental health comorbidities, as well as cognitive fluctuations; neurologists evaluated movement disorders; and neuropsychologists examined cognitive domains. A consensus diagnosis was reached through an interdisciplinary meeting, reviewing the patient's clinical evolution, examination findings, assessment scores, and test results, followed by tailored management recommendations. The diagnostic criteria for DLB were based on the Fourth Consensus Report of the DLB Consortium.^12^ For AD, we applied the International Working Group 2021 criteria^13^ and the requirements from the American Psychiatric Association^14^ for VaD. For FTD, we included patients with the behavioral variant^15^ and those with agrammatical or semantic primary progressive aphasia.^16^ The Movement Disorders Society criteria were used for PDD^17^; the information is filed in the Intellectus register with all the variables assessed, including the consensus diagnosis.

### Clinical Rating Scales Employed

All patients were systematically applied the following tools. As an outcome, cognitive screening was conducted using the Mini‐Mental State Examination (MMSE).^18^ It was validated in the Colombian population with a sensitivity and a specificity of 92.3% and 53.7%, respectively.^19^ Additionally, the Montreal Cognitive Assessment (MoCA),^20^ with a cutoff of 26 points, was validated in the Colombian population, demonstrating a sensitivity of 76% to detect mild cognitive impairment and 92.7% to detect mild dementia, and a specificity of 79.8%.^21^ Also, a neurologist applied the CDR as a general classifier of dementia.

To evaluate neuropsychiatric symptoms, psychiatrists used the Mild Behavioral Impairment Checklist (MBI‐C), which predicts dementia risk by assessing 5 domains: interest, affective symptoms, disinhibition, social norms, and delusions/hallucinations, with symptom severity rated from 1 (mild) to 3 (severe).^22^, ^23^ MBI‐C has not been validated in the Colombian population, but it has been translated into Spanish.^24^ Additionally, the Neuropsychiatric Inventory Questionnaire (NPI‐Q) was used to assess neuropsychiatric symptoms, covering 12 domains such as delusions, hallucinations, aggression, depression, and anxiety, with severity also rated from 1 (mild) to 3 (severe). The maximum score is 36, and it has been validated in multiple languages.^25^, ^26^ NPI‐Q has been validated in Spanish, showing a test–retest reliability of 0.89 and a convergence validity of 0.88.^27^ Additionally, different tests were performed by neuropsychologists, such as the patient's and caregiver's subjective memory complaint and the Frontal Systems Behavior Scale.

Basic activities of daily living were assessed by a geriatrician using the Barthel index, which evaluates a patient's independence in 10 self‐care tasks. The maximum score is 100, making it a useful tool for longitudinal follow‐up.^28^ Barthel index was validated in Spanish, reflecting good reliability and structural validity, and Cronbach's α coefficient was >0.70.^29^ Physical performance was evaluated using the Short Physical Performance Battery (SPPB), which assesses muscular function, balance, gait, strength, and endurance.^30^ SPPB is reliable and valid for assessing physical performance among older adults in Colombia, with a total test reliability of 0.87.^31^

Parkinsonism was assessed and recorded in the clinical notes and the registry through the neurological examination performed by the neurologist. Cognitive fluctuations were not recorded systematically. The registry is framed in a research project called RECOG‐HUSI, aiming to store clinical and paraclinical data based on the clinic's records for identifying patterns and improvement possibilities. It stores the information in a code to maintain confidentiality and is constantly filled by administrative personnel at the Intellectus Memory Clinic.

## Statistical Analysis

A clinician‐researcher (F.B.‐R.) searched the RECOG‐HUSI database for patients diagnosed with DLB and recorded the clinical features mentioned earlier based on the *Diagnostic and Statistical Manual of Mental Disorders*, Fifth Edition, nosology. We calculated the frequencies of each diagnostic group, and we randomly selected patients of each etiology (AD, FTD, VaD, and PDD), maintaining a maximum ratio of 4:1 compared to DLB patients to facilitate the comparability between groups but maintaining efficiency. The random selection was paired by age, but we did not include dementia severity or symptomatology duration due to the lack of data. Then, based on the paired sample, we conducted a descriptive analysis of each variable to explore the population distribution and the participants' characteristics. With this information, we evaluated the mean and proportion differences using analysis of variance (ANOVA) and standard deviation (SD), and χ^2^ test for categorical variables, and a test of equal or given proportions. We assumed associations significant at *P* < 0.05 and marked with “†” the associations significant after Benjamini–Hochberg false discovery rate correction.

Then, we performed different logistic regression models to assess the factors associated with DLB diagnosis (vs. all other etiologies) and then multinomial regression analysis to compare each diagnostic group. As independent variables, we included the clinical variables. Then we developed multinomial regression models for each diagnosis to compare DLB diagnosis with AD, FTD, VaD, and PDD. Furthermore, we assessed the correlation between variables using Pearson's analysis, and finally, we developed other multiple linear regression models assessing the relationship between cognitive, functional, and behavioral variables in the DLB population. All models included age, years of education, and CDR as control variables. *P*‐values for these models were adjusted for multiple comparisons within each model using the Benjamini–Hochberg false discovery rate procedure. We assumed associations significant at *P* < 0.05 and marked with “†” the associations significant after Benjamini–Hochberg false discovery rate correction. All analyses were performed using R Studio, version 4.2.1.

## Results

### Prevalence

Of 5518 patients, only 38 were diagnosed with DLB (0.6%). The DLB individuals were predominantly male, with a mean age of 74.5 years (SD = 8.57).

### Clinical Presentation

A comparison of DLB to other etiologies revealed significant clinical and statistical differences (Table 1). AD and FTD were assessed at more advanced stages based on the CDR. However, FTD symptoms appeared earlier, whereas AD symptoms manifested at an older age. Furthermore, differences were observed in functional, motor, and cognitive performance metrics, as indicated by MMSE, MoCA, and phonologic fluency test scores. These differences continued to be statistically significant after multiple comparisons, denoted by “†” (see Table 1).

**TABLE 1:** Sociodemographic and clinical characteristics of sample

	Dementia with Lewy bodies	Alzheimer's disease	Frontotemporal dementia	Vascular dementia	Parkinson's disease dementia	*P‐*value
	(n = 38)	(n = 312)	(n = 125)	(n = 196)	(n = 40)	
Female, n (%)	42.1%	47.4%	48.0%	46.9%	52.5%	–
Age, mean (SD)	74.5 (8.57)	78.5 (9.51)	70.0 (7.84)	77.6 (8.92)	76.4 (6.81)	–
Years of education, mean (SD)	9.77 (5.72)	9.46 (5.78)	10.6 (5.45)	9.49 (5.55)	10.3 (6.28)	0.407
CDR, mean (SD)	1.87 (1.01)	2.00 (0.763)	2.05 (0.814)	1.66 (0.718)	1.44 (0.809)	<0.001^a^
Age of first symptom, mean (SD)	70.8 (8.16)	73.9 (8.41)	63.4 (6.58)	73.2 (8.85)	69.0 (7.42)	<0.001^a^
Comorbidities (diagnosis)						
Metabolic comorbidity (%)	69.7%	61.8%	50.0%	80.8%	93.1%	<0.001^a^
CNS comorbidity, (%)	47.4%	45.5%	53.5%	46.2%	77.8%	0.100
Psychiatric comorbidity, (%)	62.1%	87.7%	81.3%	74.3%	62.1%	<0.001^a^
Functional						
Barthel, mean (SD)	70.8 (29.4)	87.2 (17.4)	78.1 (24.6)	77.0 (23.4)	72.8 (19.7)	<0.001^a^
Lawton, mean (SD)	5.32 (4.74)	6.98 (3.78)	5.92 (3.94)	5.77 (3.50)	5.49 (3.38)	0.001^a^
SPPB, mean (SD)	6.18 (4.14)	7.73 (3.15)	7.49 (3.36)	5.97 (3.46)	4.50 (3.16)	<0.001^a^
Cognitive						
MMSE, mean (SD)	18.5 (8.19)	18.8 (5.73)	17.8 (8.38)	20.5 (5.07)	21.9 (4.75)	<0.001^a^
MoCA, mean (SD)	12.4 (7.91)	11.7 (6.00)	12.0 (8.31)	13.3 (5.86)	14.8 (5.73)	0.016^a^
Neuropsychiatric symptoms						
NPI‐Q, mean (SD)	9.79 (6.02)	7.97 (5.40)	13.2 (6.62)	8.21 (5.74)	10.1 (6.17)	<0.001^a^
Delusions, mean (SD)	0.643 (1.03)	0.448 (0.884)	0.703 (1.11)	0.451 (0.955)	0.471 (0.992)	0.177
Hallucinations, mean (SD)	0.964 (1.20)	0.208 (0.635)	0.327 (0.814)	0.240 (0.691)	0.424 (0.902)	<0.001^a^
Agitation, mean (SD)	0.667 (1.04)	0.878 (1.12)	1.21 (1.22)	0.894 (1.14)	0.833 (1.11)	0.063
Depression, mean (SD)	1.32 (1.05)	1.09 (1.06)	1.21 (1.27)	1.21 (1.09)	1.60 (1.06)	0.116
Anxiety, mean (SD)	1.03 (1.19)	1.10 (1.13)	1.10 (1.30)	1.03 (1.12)	1.32 (1.17)	0.755
Euphoria, mean (SD)	0.115 (0.588)	0.0615 (0.340)	0.297 (0.729)	0.0659 (0.366)	0.0606 (0.242)	<0.001^a^
Apathy, mean (SD)	1.93 (1.13)	1.29 (1.15)	2.24 (1.11)	1.41 (1.24)	1.49 (1.17)	<0.001^a^
Disinhibition, mean (SD)	0.407 (0.931)	0.532 (0.975)	1.61 (1.28)	0.538 (0.984)	0.667 (1.05)	<0.001^a^
Irritability, mean (SD)	0.806 (1.01)	1.14 (1.10)	1.69 (1.21)	1.16 (1.13)	1.24 (1.21)	<0.001^a^
Motor disturbance, mean (SD)	0.346 (0.846)	0.399 (0.870)	0.981 (1.30)	0.247 (0.712)	0.576 (1.06)	<0.001^a^
Nocturnal behavior, mean (SD)	1.21 (1.20)	0.734 (1.02)	0.942 (1.21)	0.695 (1.06)	1.03 (1.09)	0.040^a^
Appetite disturbance, mean (SD)	1.04 (1.17)	0.730 (1.03)	1.63 (1.27)	0.636 (1.02)	0.676 (0.945)	<0.001^a^
MBI‐C, mean (SD)	25.9 (13.6)	21.4 (15.2)	35.6 (17.1)	23.1 (15.8)	26.4 (16.4)	<0.001^a^
Motivation, mean (SD)	9.03 (5.56)	6.43 (5.19)	10.9 (5.69)	7.05 (5.44)	7.89 (5.11)	<0.001^a^
Affection, mean (SD)	7.66 (4.57)	5.52 (4.54)	6.76 (4.62)	6.36 (5.21)	8.00 (4.41)	0.004^a^
Behavior, mean (SD)	6.16 (5.70)	6.84 (5.78)	11.2 (7.05)	6.83 (5.88)	6.69 (6.68)	<0.001^a^
Social norms, mean (SD)	1.07 (1.80)	1.67 (2.43)	4.13 (3.99)	1.89 (2.91)	2.03 (3.34)	<0.001^a^
Thought and perception content, mean (SD)	2.87 (3.30)	1.21 (2.18)	2.18 (3.15)	1.18 (2.35)	1.80 (3.45)	<0.001^a^
Yesavage, mean (SD)	6.10 (4.34)	3.61 (3.10)	4.21 (3.62)	5.39 (3.95)	6.30 (3.67)	<0.001^a^
SMCp, mean (SD)	19.5 (11.1)	14.5 (11.4)	12.1 (9.40)	17.5 (10.2)	19.1 (9.65)	<0.001^a^
SMCf, mean (SD)	23.1 (10.2)	30.3 (8.31)	29.4 (10.9)	26.0 (9.42)	25.3 (9.93)	<0.001^a^
FRSB, mean (SD)	133 (15.5)	124 (32.8)	144 (30.8)	134 (28.8)	135 (22.5)	<0.001^a^
Stop Bang, mean (SD)	7.43 (5.82)	7.27 (5.39)	6.21 (5.86)	8.48 (5.62)	9.96 (7.02)	0.016^a^
MNA, mean (SD)	9.12 (2.80)	11.3 (2.22)	11.3 (2.52)	11.9 (1.87)	10.7 (2.26)	<0.001^a^
Gastrointestinal symptoms, n (%)	40.0%	7.7%	31.0%	27.9%	20.6%	<0.001^a^

*Note*: *P*‐values were adjusted for multiple comparisons within each model using the Benjamini–Hochberg false discovery rate procedure.

^a^ Denotes associations significant at *P* < 0.05 after Benjamini–Hochberg false discovery rate correction within each comparison.

Abbreviations: SD, standard deviation; CDR, clinical dementia rating; CNS, central nervous system; SPPB, Short Physical Performance Battery; MMSE, Mini‐Mental State Examination; MoCA, Montreal Cognitive Assessment; NPI‐Q, Neuropsychiatric Inventory Questionnaire; MBI‐C, Mild Behavioral Impairment Checklist; SMCp, subjective memory complaint of patient; SMCf, subjective memory complaint of familiar; FRSB, Frontal Systems Behavior Scale; MNA, Mini Nutritional Assessment.

Differences regarding neuropsychiatric symptoms were observed among the various dementia etiologies. Visual and auditory hallucinations, as well as disturbances in nocturnal behavior, were more prevalent in DLB and PDD patients. Variability in symptomatology was statistically significant (χ^2^ = 106.428, *P* <0.001) (see Table 2). MBI‐C scores were higher in FTD and DLB but lower in AD and VaD. Motor disturbances were more pronounced in FTD and PDD (see Table 1).

**TABLE 2:** Sensoperceptive symptoms by etiology, n (%)

	Dementia with Lewy bodies	Alzheimer's disease	Frontotemporal dementia	Vascular dementia	Parkinson's disease dementia	Total (%)
Absence	13 (35)	254 (82.5)	86 (71.1)	160 (82.9)	24 (60)	537 (0.85)
Visual	22 (60)	25 (8)	21 (17)	18 (9)	12 (29)	98 (0.16)
Auditory	1 (2.7)	6 (1.9)	5 (4.1)	2 (1)	2 (5)	16 (0.01)
Tactile	1 (2.7)	1 (0.3)	0	1 (0.5)	0	3 (0.002)
Sense of presence	1 (2.7)	2 (0.6)	2 (1.7)	1 (0.5)	1 (2.5)	7 (0.005)

*Notes*: χ^2^ = 106.428; *P‐*value <0.001.

### Prevalence of Core DLB Symptoms across Other Dementias

The presence of core and suggestive symptoms of DLB was observed in individuals diagnosed with AD, FTD, VaD, and PDD. Particularly, nearly half of the patients exhibited at least 1 core DLB feature, whereas up to 22% presented with 2 core symptoms, and 12% presented with 3 core features at the time of assessment. Among the core features, parkinsonism was the more frequent compared to visual hallucinations and rapid eye movement sleep behavior disorder. Suggestive features like recurrent falls and constipation were also evaluated, and there was a higher frequency of recurrent falls than constipation (see Table 3).

**TABLE 3:** Dementia with Lewy bodies symptoms in other etiologies, n (%)

	Dementia with Lewy bodies	Alzheimer's disease dementia	Frontotemporal dementia	Vascular dementia	Parkinson's disease dementia
Number of core features					
None	13 (34.2)	176 (56.4)	63 (50.4)	63 (32.1)	11 (27.5)
One	6 (15.8)	115 (36.9)	49 (39.2)	117 (59.7)	15 (37.5)
Two	9 (23.7)	16 (5.1)	10 (8)	12 (6.1)	9 (22.5)
Three	10 (26.3)	5 (1.6)	3 (2.4)	4 (2)	5 (12.5)
Core and suggestive features					
Parkinsonism^a^	21 (58)	128 (41)	53 (42)	129 (66)	29 (72)
Visual hallucinations	22 (60)	25 (8)	21 (17)	18 (9)	12 (29)
RBD	14 (48.3)	9 (3.1)	7 (6.7)	8 (4.6)	9 (27.3)
Recurrent falls	9 (32.1)	44 (15.4)	19 (21.3)	36 (23.5)	7 (21.9)
Constipation	16 (40)	25 (8)	39 (31)	55 (28)	8 (21)

*Note*: Results are presented as n (%): number (percentage).

^a^ Presence of parkinsonism is reported based on clinical findings at the Intellectus evaluation.

Abbreviation: RBD, rapid eye movement sleep behavior disorder.

### Characteristics within the Different Diagnosis

**FIG. 1:**
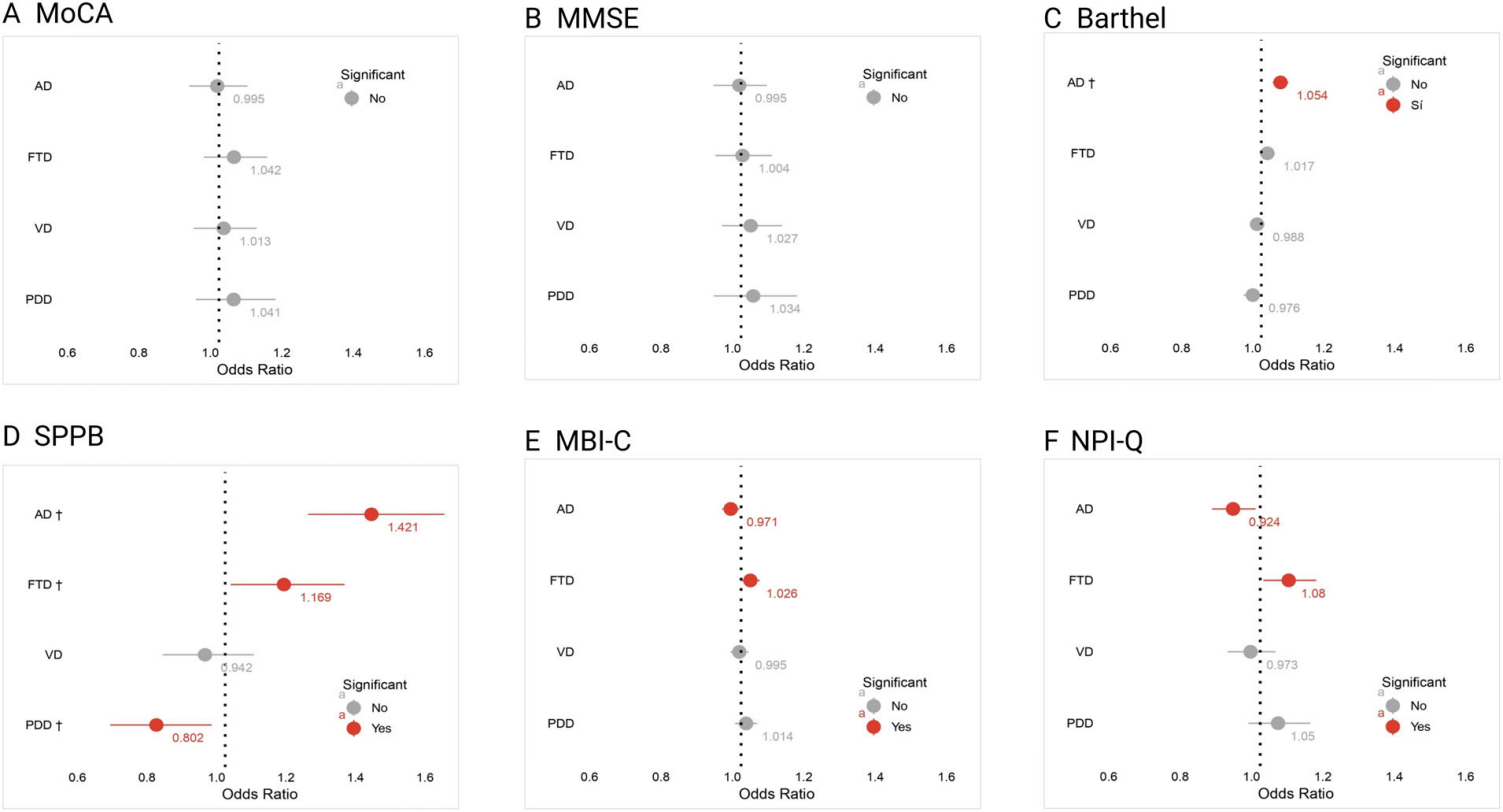
Odds ratios of factors associated to dementia with Lewy bodies diagnosis compared with other etiologies according tests results. (A). MoCA: Montreal Cognitive Assessment (B). MMSE: Mini‐Mental State Examination (C). Barthel index (D). SPPB: Short Physical Performance Battery (E). MBI‐C: Mild Behavioral Impairment Checklist (F). NPI‐Q: Neuropsychiatric Inventoy Questionnaire. AD, Alzheimer's disease; FTD, frontotemporal dementia; OR, odds ratio; PDD, Parkinson's disease dementia; SPPB, Short Physical Performance Battery; VaD, vascular dementia. All models include age and years of education as control variables. Red lines denote *P*‐value <0.05, and “†” denotes associations significant at *P* < 0.05 after Benjamini–Hochberg false discovery rate correction within each model.

In the logistic regression analysis, lower Barthel and SPPB scores were associated with a higher likelihood of a DLB diagnosis compared to other types of dementia. Stratifying by specific diagnoses, compared to AD, higher Barthel and SPPB scores, along with lower NPI‐Q scores, increased the likelihood of a DLB diagnosis. In contrast to FTD, lower MBI‐C and NPI‐Q scores were significantly associated with DLB. Compared to VaD and PDD, higher MMSE scores were linked to greater odds of a DLB diagnosis. After correction for multiple comparisons, Barthel continued to be statistically significant when DLB diagnosis was compared with AD, and the SPPB was significant when DLB was compared with AD, FTD, and PDD (see Table 4 and Figure 1).

**TABLE 4:** Factors associated to dementia with Lewy bodies diagnosis compared with other etiologies

	MoCA		MMSE		Barthel		SPPB		MBI‐C		NPI‐Q	
	OR	*P*‐value	OR	*P*‐value	OR	*P*‐value	OR	*P*‐value	OR	*P*‐value	OR	*P*‐value
AD	0.995	0.903	0.995	0.886	1.054	<0.001^a^	1.421	<0.001^a^	0.971	0.018	0.924	0.019
FTD	1.042	0.343	1.004	0.921	1.017	0.108	1.169	0.029^a^	1.026	0.049	1.080	0.026
VaD	1.013	0.767	1.027	0.515	0.988	0.256	0.942	0.401	0.995	0.721	0.973	0.441
PDD	1.041	0.467	1.034	0.556	0.976	0.064	0.802	0.017^a^	1.014	0.391	1.050	0.248

*Notes*: *P*‐values were adjusted for multiple comparisons within each model using the Benjamini–Hochberg false discovery rate procedure. All models include age and years of education as control variables.

^a^ Denotes associations significant at *P* < 0.05 after Benjamini–Hochberg false discovery rate correction within each model.

Abbreviations: MoCA, Montreal Cognitive Assessment; MMSE, Mini‐Mental State Examination; SPPB, Short Physical Performance Battery; MBI‐C, Mild Behavioral Impairment Checklist; NPI‐Q, Neuropsychiatric Inventory Questionnaire; OR, odds ratio; AD, Alzheimer's disease; FTD, frontotemporal dementia; VaD, vascular dementia; PDD, Parkinson's disease dementia.

### Cognitive, Functional, and Behavioral Performance in DLB Population

The correlation analysis revealed statistically significant associations: MoCA correlated positively with MBI‐C, Barthel, and SPPB (r = −0.38, r = 0.64, r = 0.55, respectively). Similarly, MMSE showed significant correlations with Barthel (r = 0.7) and SPPB (r = 0.52). Finally, in the linear regression analysis, Barthel and SPPB had a positive association with MoCA and MMSE, and behavioral variables did not show a significant association with others (see Table S1).

## Discussion

This study provides a comprehensive analysis of the prevalence and clinical characteristics of DLB in a large Latin American memory clinic, contributing to the limited body of literature on DLB in this region.^6^ The frequency observed in our study was significantly low, with only 0.6% of patients diagnosed with DLB in a referral memory clinic. This starkly contrasts with the global prevalence rates, which range between 3.2% and 7.1% of all diagnosed dementias, and as high as 24.4% in some prevalence studies.^3^ The need arises to investigate whether it is due to a lower prevalence or because DLB remains underrecognized or misidentified in this setting.^5^

We believe the primary reason for the low observed frequency of DLB diagnosis is underdiagnosis. Supporting this, a significant proportion of patients in the non‐DLB groups exhibited ≥2 core clinical features, even without the assessment of fluctuating cognition, with a high proportion presenting with at least 1 core feature. According to current diagnostic criteria, a patient with dementia and at least 2 core clinical features can be diagnosed with probable DLB rather than AD, unless biomarker evidence or other clinical factors indicate otherwise.^12^

Several factors may contribute to the possible underdiagnosis of DLB in our population. First, its clinical presentation is highly variable, with symptoms such as cognitive fluctuations, visual hallucinations, and motor impairments overlapping with other neurodegenerative conditions like AD and VaD.^8^, ^32^ This overlap creates a significant diagnostic challenge, particularly in settings with limited access to specialized diagnostic tools and trained personnel, making it difficult for clinicians to distinguish DLB from other dementias. Furthermore, similar trends have been observed in studies from high‐income countries, such as the United States,^33^ underscoring the even greater diagnostic barriers faced by LMICs, although there is reported to be a higher prevalence of dementia in underrepresented racial and ethnic groups compared to people identifying as White, supported as they have more risk factors for developing dementia.^34^ These challenges added to the dementia‐related stigma, reducing the chances of seeking assistance from the physician and, therefore, making prompt diagnosis difficult.^35^ Also, cultural perceptions and beliefs play a pivotal role in the recognition and diagnosis of dementia in Latin America. In Colombia and other countries in the region, cognitive decline is often perceived as a normal part of aging, which may delay care‐seeking behavior and contribute to late‐stage diagnoses.^36^ Moreover, stigma associated with dementia can discourage individuals and families from discussing symptoms or seeking formal evaluation, further complicating early detection, and may contribute to the underrepresentation of DLB, jointly with other cultural barriers that should be studied in depth and foster culturally sensitive public health.

Additionally, a snowball effect may be occurring, where a low diagnosis rate reduces awareness and training about the disease. This leads to more missed cases, further lowering the reported prevalence and perpetuating the cycle. This also might make it difficult to recruit patients for research purposes^11^ and represent a lower frequency than in the community population. Another possible cause could be lower referral rates of DLB cases to memory clinics such as ours, as patients are often managed in primary care without a clear dementia diagnosis or misclassified as AD or PD. It is also possible that some individuals with DLB—particularly those whose initial presentation involved parkinsonian features—may have been evaluated in movement disorder clinics rather than memory clinics.^37^ Because our data are limited to a memory clinic setting, this referral pattern could have contributed to an underrepresentation of DLB cases in our sample. Additionally, challenges related to DLB diagnosis arise when differences in the relationship between genetics and environment in Colombia are considered. On one hand, the ancestry in the region has been studied less in DLB, as in FTD, and could explain a different prevalence.^38^, ^39^ On the other hand, there are environmental characteristics, such as cardiometabolic and socioeconomic risk factors, that are more prevalent in LMICs like Colombia and are related to neurodegenerative diseases, mainly to etiologies like AD and VaD.^40^, ^41^

Our study underscores distinct clinical differences between DLB and other neurodegenerative dementias. Compared to AD and FTD, DLB patients exhibited more severe neuropsychiatric symptoms, including hallucinations and behavioral disturbances, aligning this finding with previous research.^32^, ^42^ Accurately distinguishing across dementia subtypes remains a challenge, particularly in both the early and late stages of the disease.^43^, ^44^ Therefore, accurate and early diagnosis is essential to improve personalized management and to prevent inappropriate use of medication.^7^

Individuals with higher MMSE scores had greater odds of being diagnosed with DLB in contrast to previous reports.^45^ However, this can be explained by the earlier‐stage identification of DLB patients in our study. In addition, both DLB and PDD exhibited lower NPI‐Q and MBI‐C scores than FTD, which aligns with previous findings, and even the description of more psychiatric comorbidity in FTD.^46^ Schwertner et al.^47^ highlighted that at least 1 neuropsychiatric symptom was present in 92.3% of individuals with dementia. Among these, FTD had the highest mean total NPI‐Q score, followed by DLB, AD, PDD, and VaD. Consistent with previous literature,^47^ our results showed a higher risk of hallucinations and a lower risk of euphoria in DLB. However, our findings differ in that FTD appears to have a greater risk of delusions.

Although this study was conducted in a single center in Colombia, we acknowledge that Latin America is a culturally, socioeconomically, and genetically diverse region. Therefore, our findings should not be overgeneralized to the entire continent. Although the data on DLB are limited, our results contribute valuable insights that may reflect some shared regional challenges, particularly those related to underdiagnosis, access to care, and limited use of standardized diagnostic tools. Therefore, one of the major strengths of this study is its use of a large, well‐characterized cohort, with a systematic assessment, from one of the largest memory clinics in Latin America. To the best of our knowledge, this is the first work that compares the symptomatology between etiologies and assesses the presence of core and suggestive symptoms for DLB in a specialized Latin American center. We consider that our results call for fostering an active detection of DLB.

The interdisciplinary approach to clinical assessment, involving geriatricians, psychiatrists, neurologists, and neuropsychologists, ensured a comprehensive evaluation of patients. Additionally, the use of standardized diagnostic criteria for DLB^12^ enhances the validity of the diagnoses. The inclusion of other dementia subtypes (AD, FTD, VaD, and PDD) as comparison groups further strengthens the study by allowing for a detailed exploration of clinical and neuropsychiatric differences between these conditions. It is worth mentioning that parkinsonism was not systematically documented in 28% of the cases during the memory clinic assessment. This may be due to 1 or more of the following reasons^1^: motor symptoms were already stabilized or masked due to ongoing antiparkinsonian treatment at the time of the cognitive evaluation^2^; the primary focus of the clinical encounter was cognitive rather than motor assessment, leading to underreporting in the memory clinic record; or^3^ motor signs were not overtly present or clinically significant during that specific consultation.

However, this study has several limitations. First, its cross‐sectional design limits our ability to conclude disease progression and longitudinal changes in symptoms and cognitive function, which is also relevant in differentiating etiologies. Second, as we do not have symptomatology progression and patients are at different stages, the neurodegeneration level and comorbidities can overlap with symptomatology between groups, potentially contributing to the underdiagnosis or misclassification of DLB in clinical practice. Further studies should focus on comorbidities that are different in Latin America from the rest of the world. Third, although our study captures the age at which symptoms first emerged, the absence of detailed data on the nature of the initial symptom limits our ability to examine early clinical differentiation between dementia subtypes. Further, dementia severity can be different between patients at the moment of diagnosis, limiting the comparability within groups. Future research with prospective designs and more granular symptom timelines would be essential to better understand how specific early features may inform diagnosis and disease classification. Fourth, as the study was conducted in a single memory clinic, and statistical methods limit the description of this population, its findings may not be generalizable to other regions of Latin America. Nevertheless, due to the limited number of specialized centers for neurocognitive disorders in the region, our findings provide valuable insights. Finally, we did not systematically assess cognitive fluctuations, despite their significance as a core diagnostic feature. The absence of biomarkers such as dopamine transporter single photon emission computed tomography (DaT‐SPECT) and positron emission tomography (PET) with Fluorodopa 18F (FDOPA) or blood and cerebrospinal fluid biomarkers may reduce diagnostic accuracy. Considering the variability in health‐care access and diagnostic expertise across the region, the challenges of underdiagnosis and misidentification of DLB are likely even more pronounced in less‐specialized settings. In Colombia, where access to definitive confirmation tests such as DaT‐SPECT and PET with FDOPA is limited, diagnoses rely primarily on clinical assessments. Future studies employing tailored methodologies are needed to accurately determine the prevalence of DLB in community settings across Colombia.

Therefore, in line with Bayram et al.^34^ recognizing and diagnosing DLB will improve the representation of this pathology and will allow more accurate data, like prevalence and risk factors, to be approached from a clinical and research setting. Additionally, the approach should use culturally adapted tools such as neuropsychological tests.

This study offers valuable insights into the prevalence and clinical characteristics of DLB in a major Latin American memory clinic, highlighting variations in neuropsychiatric and motor symptoms across dementia subtypes. Our findings suggest that contextual factors may play a role in the underdiagnosis of DLB, emphasizing the need for greater awareness and improved diagnostic strategies in the region. Strengthening health‐care training, integrating advanced diagnostic tools, and enhancing diagnostic accuracy are essential steps toward better identification and management of DLB, ultimately improving patient care and quality of life.

## Author Roles

(1) Research project: A. Conception, B. Organization, C. Execution; (2) Methodological analysis: A. Design, B. Execution, C. Review and critique; (3) Manuscript preparation: A. Writing of the first draft, B. Review and critique, C. Review and approval.

F.B.‐R.: 1A, 1B, 1C, 2A, 2B, 2C, 3A, 3C

C.C.‐G.: 1A, 1B, 2C, 3B, 3C

J.M.S.‐E.: 2C, 3B, 3C

S.S.‐L.: 1A, 1C, 2B, 3A, 3C

M.G.B.: 1A, 1B, 2C, 3B, 3C

D.A.: 1A, 2C, 3B, 3C

## Disclosures

**Ethical Compliance Statement:** The research protocol was approved by the Institutional Research Ethics Committee of San Ignacio University Hospital and Pontificia Universidad Javeriana (act no. 04/2024). Informed patient consent was not necessary for this work. We confirm that we have read the journal's position on issues involved in ethical publication and affirm that this work is consistent with those guidelines.

**Funding Sources and Conflicts of Interest:** This study was funded by the Norwegian Health Association and also by the National Institute for Health Research (NIHR) Biomedical Research Centre at South London and Maudsley NHS Foundation Trust and King's College London. The authors declare that there are no conflicts of interest relevant to this work.

**Financial Disclosures for the Previous 12 Months:** None.

## Supporting information


**Table S1.** Associated factors with cognitive, functional, and behavioral performance in dementia with Lewy bodies patient.

## Data Availability

The data that support the findings of this study are available on request from the corresponding author. The data are not publicly available due to privacy or ethical restrictions.
