# Supplementary material for: Low Frequency of Dementia with Lewy Bodies Diagnosis in a Colombian Memory Clinic
Source: Mov Disord Clin Pract. 2025 Sep 8;13(2):442–51. doi: 10.1002/mdc3.70345 (PMC12911454; doi:10.1002/mdc3.70345)
Supplement: Supplementary file 1 — Table S1. Associated factors with cognitive, functional, and behavioral performance in dementia with Lewy bodies patient. [file MDC3-13-442-s001.docx]

| **Supplementary material 1.** Associated factors with cognitive, functional and behavioral performance in Lewy Body Dementia patients | | | | | | | | | | | | |
| --- | --- | --- | --- | --- | --- | --- | --- | --- | --- | --- | --- | --- |
|  | Cognition | | | | Functionality | | | | Behavioral | | | |
|  | MoCA | p value | MMSE | p value | Barthel | p value | SPPB | p value | MBI-C | p value | NPI-Q | p value |
|  |  |  |  |  |  |  |  |  |  |  |  |  |
| MoCA |  |  |  |  | 3,171 | <0,001 | 0,315 | 0,009 | ´-0,125 | 0,140 | ´-0,028 | 0,437 |
| MMSE |  |  |  |  | 2,700 | <0,001 | 0,275 | 0,009 | ´-1,064 | 0,127 | ´-0,245 | 0,417 |
| Barthel | 0,134 | <0,001 | 0,186 | <0,001 |  |  |  |  | ´-0,561 | 0,18 | ´-0,037 | 0,843 |
| SPPB | 0,717 | 0,009 | 0,878 | 0,009 |  |  |  |  | ´-0,428 | 0,201 | ´-0,034 | 0,819 |
| MBI-C | ´-0,114 | 0,170 | ´-0,139 | 0,201 | ´-0,591 | 0,139 | ´-0,082 | 0,127 |  |  |  |  |
| NPI-Q | ´-0,043 | 0,843 | ´-0,066 | 0,819 | ´-0,869 | 0,437 | ´-0,123 | 0,417 |  |  |  |  |
| MoCA: Montreal Cognitive Assessment; MMSE: Mini-Mental State Examination; SPPB: Short Physical Performance Battery; MBI-C: Mild Bevavioral Impairment checklist; NPI-Q: Neuropsichyatric Inventoy questionnaire. All models include age and years of education as control variables. | | | | | | | | | | | | |
